# How I do it: Surgical interruption of high-flow dural arteriovenous fistulas at the foramen magnum region

**DOI:** 10.1007/s00701-024-06370-x

**Published:** 2024-11-20

**Authors:** Yusuke Egashira, Masaki Kumagai, Yukiko Enomoto, Tsuyoshi Izumo

**Affiliations:** Department of Neurosurgery, Gifu University Graduate School of Medicine, 1-1 Yanagido, Gifu, 501-1194 Japan

**Keywords:** Dural arteriovenous fistula, Foramen magnum region, Surgical interruption, Transarterial embolization

## Abstract

**Background:**

Dural arteriovenous fistulas (dAVFs) in the foramen magnum region (FMR) are rare entity of dAVFs. There is no established treatment for FMR-dAVFs owing to their rarity and anatomical complexity. Herein, we report cases of high-flow dAVFs located at the posteromedial part of the FMR that were successfully treated by surgical interruption.

**Methods:**

We demonstrated the surgical procedures for the interruption of high-flow FMR-dAVF with representative images and videos. In both cases, endovascular transarterial embolization was performed prior to surgical interruption.

**Conclusion:**

As this type of FMR-dAVF has high-risk clinical features, curative surgical treatment is highly desirable.

**Supplementary information:**

The online version contains supplementary material available at 10.1007/s00701-024-06370-x.

## Relevant surgical anatomy

Dural arteriovenous fistulas (dAVFs) located in the foramen magnum region (FMR) is a rare entity of dAVFs, and the incidence of FMR-dAVFs is reportedly between 1.5 and 4.2% of intracranial dAVFs [2]. FMR is anatomically complex because many adjacent venous systems are involved in this region. Caton et al. [2] was reviewed the FMR-AVFs and classified them into four subtypes. However, they were mainly focused on the dAVFs located on the anterolateral part of the FMR, known as hypoglossal canal dAVF or anterior condylar confluence (ACC) dAVF. Another major subtype of FMR-dAVF presents a shunt structure similar to a spinal dAVF; in these cases, the shunted point is located within the dura around the upper cervical nerve root [8]. In contrast, high-flow dAVFs with exclusive venous reflux via occipital bridging veins (BVs) without sinus draining located at the posteromidline part of the FMR should be considered a different form from the aforementioned FMR-dAVFs. According to a previous large case series, this posteriorly located high-flow dAVFs with Borden type III [1], Cognard type IV [4] features seemed to comprise only 11% of FMR-dAVFs [2]. Despite the high-risk features for intracranial hemorrhage and congestive myelopathy, optimal treatment strategies have not been well described [2, 3].

**Fig. 1:**
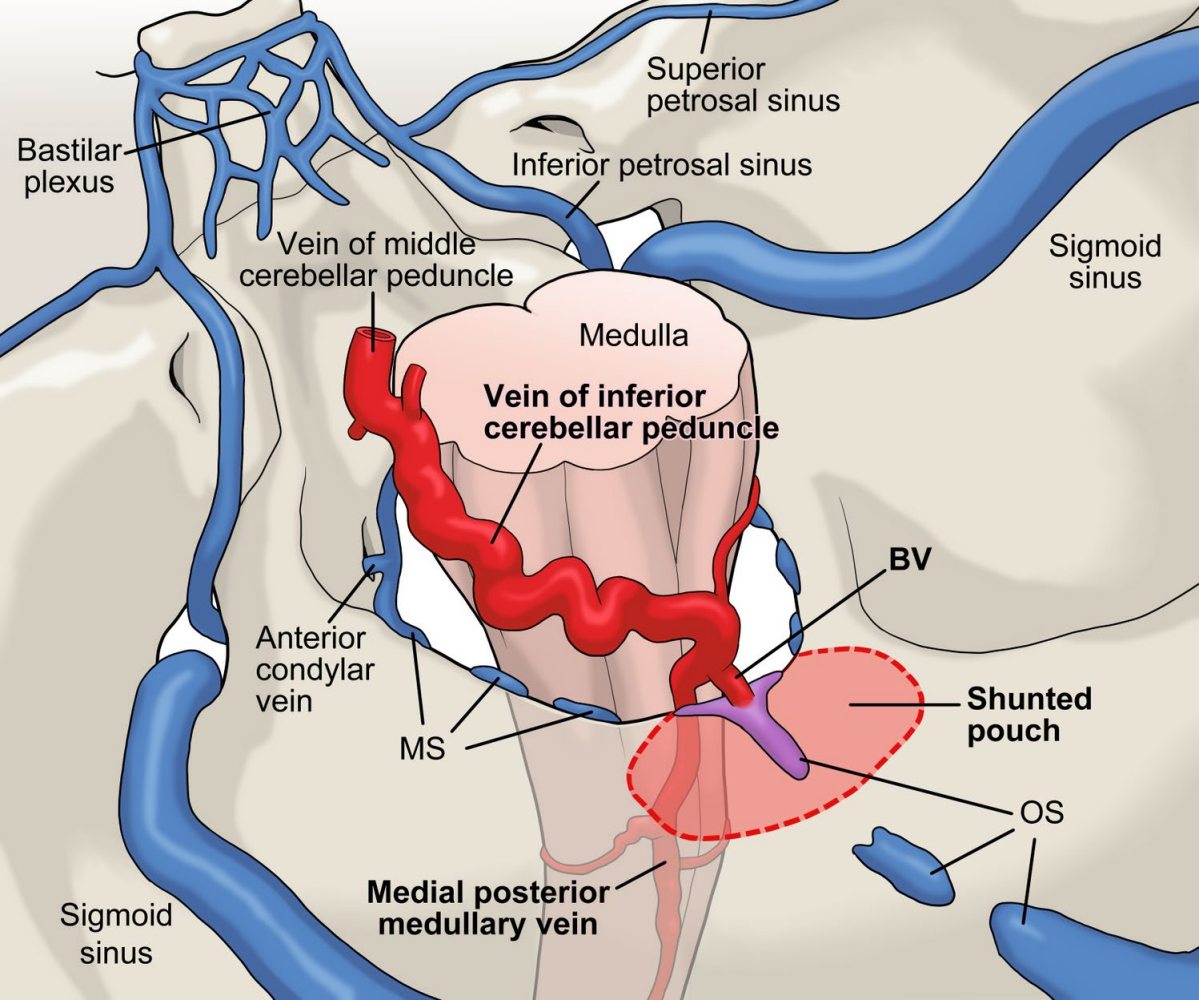
Schematic drawing of the dural arteriovenous fistula (dAVF) at the posterior medial part of the foramen magnum region (FMR). The occipital sinus (OS) and the marginal sinus (MS) are anatomically variable and usually have inconsistent structures. The shunted pouch is located around the scattered structure of the OS and MS. The bridging vein (BV), which communicates between the FMR and the vein of inferior cerebellar peduncle or the medial posterior medullary vein rarely connects to the scattered OS or MS in the posterior medial portion. This BV works as the single and main drainer of the high-flow dAVF located in the posterior medial part of the FMR

The marginal sinus (MS) runs along the rim of the foramen magnum as an intradural sinus. The MS usually has an inconstant structure, and communicates laterally with the ACC via the condylar veins, and posteromedially with the occipital sinus (OS) [9]. Both the MS and OS are usually regress and become inconsistent structures in adults, and many anatomical variations exist [10]. At the posterolateral part of the FMR, a few BVs that connect the MS and the medial posterior medullary vein or the vein of inferior cerebellar peduncle [5]. The rare variant of BV has been reported to be directly connected to OS at the midline of the FMR [6]. This anatomical variant of BV may only become apparent in pathological conditions; the BV works as a drainer of hypervascular tumors or high-flow dAVFs, as described here. The inconsistent and varied structures of the OS and MS may also be involved in this unique anatomical feature of the posterior midline located FMR-dAVFs. A schematic drawing of this type of FMR-dAVFs is shown in Fig. 1.

## Description of the technique

**Fig. 2:**
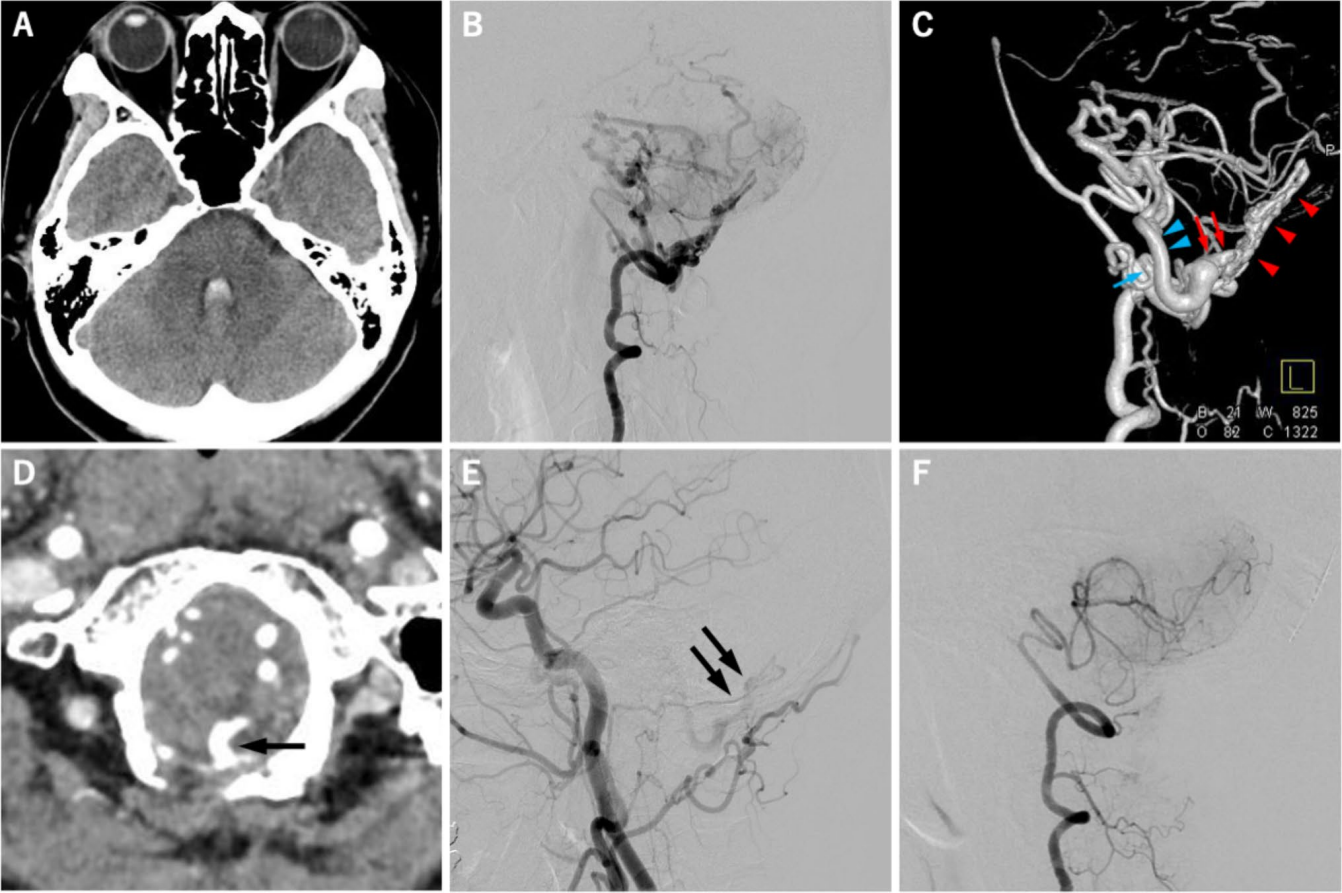
A 60-year-old female presented sudden onset of severe headache. The computed tomography (CT) image on admission showed the small amount of subarachnoid hemorrhage (SAH) and intraventricular hemorrhage (IVH) in the fourth ventricle (**a**). The lateral view of right vertebral angiogram (VAG) revealed high-flow dural arteriovenous fistula (dAVF) at foramen magnum region (FMR) with marked dilatation of posterior fossa veins (**b**). Left lateral view of 3-dementional rotational right VAG demonstrated shunted pouch on the surface of dura matter (red arrowhead) and the bridging vein (BV: red arrow) that works as a single drainer of dAVF and connected to vein of inferior cerebellar peduncle (blue arrowhead) and to posterior medial medullary vein (blue arrow) (**c**). The BV (arrow) that connecting FMR and posterior fossa veins was demonstrated by the source image of CT angiography (d). The lateral view of left carotid angiogram after endovascular transarterial embolization (TAE) of main feeders showed a small amount of residual shunt flow from the dural branches of ascending pharyngeal artery (**e**). The lateral view of right VAG obtained two weeks after surgical interruption showed the complete disappearance of high-flow FMR-dAVF (**f**)

Here, we report two cases of high-flow dAVFs located at the posterior midline part of the FMR. Both patients presented with a subarachnoid hemorrhage (SAH). The vascular anatomy was similar in both cases. Case 1 involved a 60-year-old female who presented with SAH, predominantly in the cerebellomedullary cistern and fourth ventricle, was transferred to our institution. Detailed images of case 1 are presented in Fig. 2, and the detailed operative procedures are demonstrated in the supplementary video. Contrast-enhanced CT scan showed a remarkable dilatation of leptomeningeal veins in the posterior cranial fossa, suggesting high-flow arteriovenous shunts. The 4-vessel digital subtraction angiography (DSA) revealed a high-flow dAVF in the midline-posterior part of the FMR. The dAVF was primarily fed by bilateral posterior meningeal arteries, predominantly on the right side. The shunted blood flow was drained via the BV connected to the posterior medial part of the FMR and diverted into the vein of inferior cerebral peduncle upward and the medial posterior medullary vein downward. Urgent endovascular transarterial embolization (TAE) was performed three days after symptom onset to reduce the shunted blood flow and risk of recurrent bleeding. Two feeders, bilateral posterior meningeal arteries (right posterior meningeal artery was considered as a main feeder), were embolized using 25% of n-butyl-2-cyanoacrylate (Histoacryl®: B Braun Aesculap AG, Tuttlingen, Germany). A significant reduction in shunted flow was achieved, and the patients recovered without any neurological deficits; however, a followed-up left carotid angiogram obtained three months later revealed a small amount of residual shunt flow from the ascending pharyngeal artery. Thus, the patient underwent surgical interruption of dAVF four months after the initial ictus.

The patient was placed in the prone position with the neck flexed and head fixed using skull clamps. A midline skin incision between 2 cm above the inion and the spinous process of the second cervical vertebra was made. A 5 cm-width, 4 cm-height of midline suboccipital craniotomy was performed with opening the foramen magnum, and a laminectomy of the first cervical vertebra (C1) was added; then, the occipital dural surface was widely exposed. A paramedian incision of the dura was started from the caudal and then incised upward to avert the shunted pouch that located at the medial portion of the FMR. This allowed for the early and safe identification of the main drainer. Abnormal vascular tangles were found around the main drainer on the back surface on the dura mater. A single BV that worked as a drainer was gently denuded to ensure sufficient length for performing interruption procedures. Refluxed blood flow into the main drainer was confirmed by using indocyanine green (ICG) videoangiography. A temporary aneurysm occlusion clip was placed, and the disappearance of the shunted blood flow into the venous system was confirmed by ICG videoangiography. The abnormal vascular tangle on the back surface of the dura and the drainer were well coagulated, and the drainer was then cut. Complete disappearance of venous reflux into the leptomeningeal veins was confirmed using Doppler ultrasonography. The shrunk dura by electrocoagulation was compensated by the artificial dura (DuraGen®: Integra LifeSciences, Princeton, NJ, USA), following which the wound was closed in the usual manner. Postoperative DSA performed immediately after the operation showed the complete disappearance of the dAVF.

**Fig. 3:**
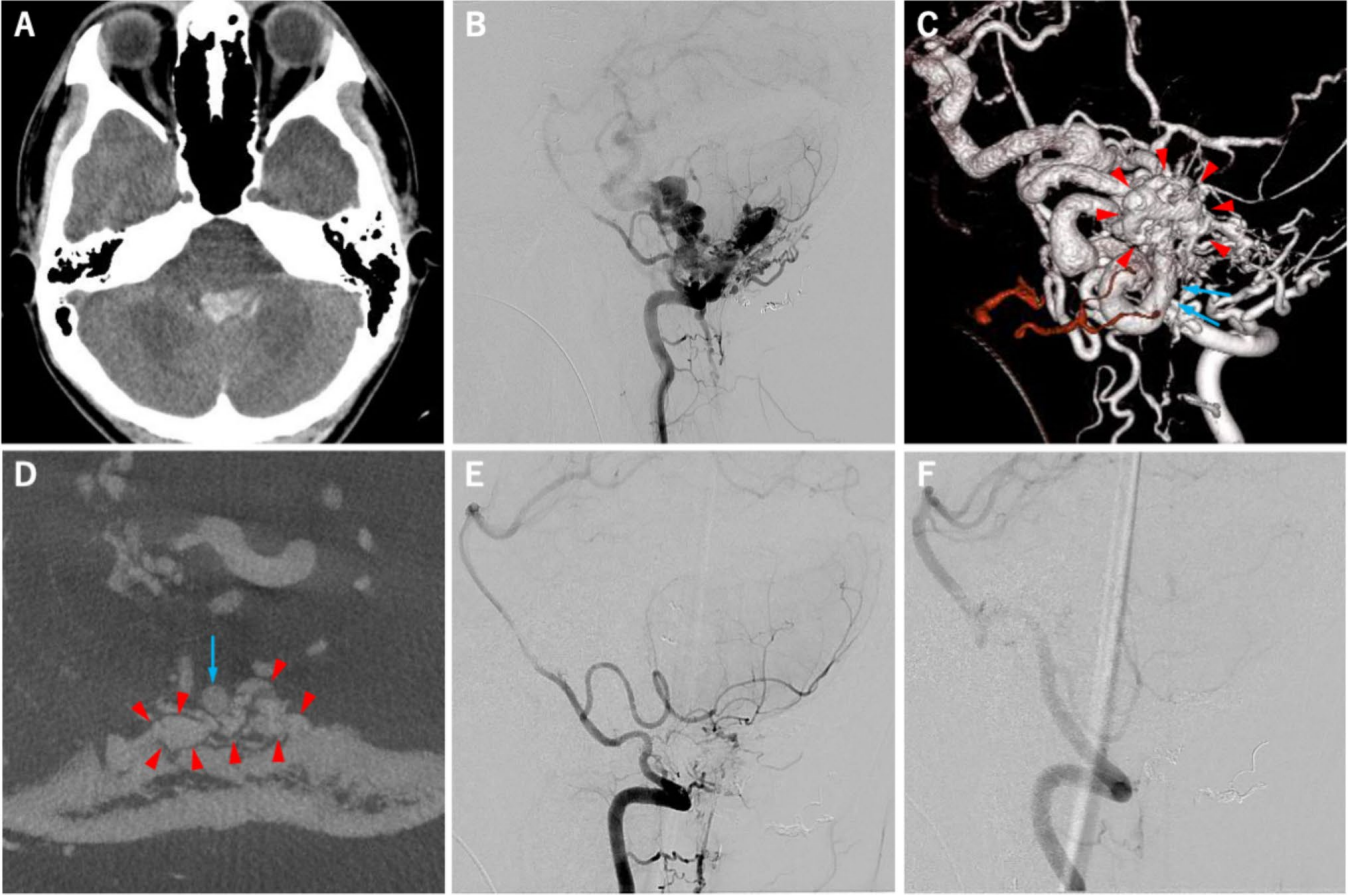
A 17-year-old male presented sudden headache followed by consciousness disturbance. The CT image on admission showed the SAH and dense IVH in the fourth ventricle (**a**). The lateral view of right vertebral VAG revealed high-flow dAVF at FMR with the remarkable dilatation of the posterior fossa veins (**b**). Posterior-anterior view of 3-dementional rotational right VAG demonstrated shunted pouch on the dura matter (red arrowhead) and the BV (blue arrow) that working as a single drainer of dAVF (**c**). The BV (blue arrow) and shunted pouch on the inner surface of dura matter (red arrowhead) was demonstrated by the axial image of cone-beam CT (**d**). This patient underwent 2-session of TAEs followed by surgical interruption via midline-suboccipital craniectomy with the same fashion as in case 1. The lateral images of right (**e**) and left (**f**) VAG obtained after surgery showed the complete disappearance of high-flow dAVF

The other patient (case 2) was a 17-year old male presented with SAH. Detailed images of this case are shown in Fig. 3. The patient also underwent 2-session of endovascular TAE, followed by surgical interruption of FMR-dAVF. In this case, the amount of residual shunt flow after endovascular TAE was greater than that in case 1, which resulted in 1,700 mL of intraoperative bleeding that mainly occurred during craniotomy from the surface of the dura.

In both case 1 and 2, repeated DSA was performed two and three weeks after the operation, respectively, and no residual or recurrence of the dAVF were noted. In addition, followed-up magnetic resonance angiography conducted up to 42 and 17 months after the operation in case 1 and 2, respectively, showed no recurrence of the dAVF.

## Indication

Because this type of FMR-dAVFs anatomically presents as Borden type III and Cognard type IV, and is clinically considered to be at high-risk for intracranial hemorrhage or congestive myelopathy, curative surgical treatment would be highly desirable. [7]

## Limitations

Anatomical details of this type of FMR-dAVFs have rarely been reported. Furthermore, suggested treatment strategies have not yet been reported. Theoretically, surgical interruption of the drainer seemed to be the best treatment procedure, whereas curative endovascular TAE using a liquid embolic agent is a potential alternative.

## How to avoid complications

To avoid massive bleeding from the dural surface during craniotomy, the endovascular TAE must be considered before direct surgery. If somewhat major bleeding from the dural surface occur, the collagen-bound fibrin sealant, ready-to-use membrane-like adhesive product, TachoSil® (CSL Behring, King of Prussia, PA, USA) is very useful to achieve hemostasis.

## Specific perioperative considerations

The optimal duration between endovascular TAE and craniotomy has not been established. However, if there are considerable amount of residual refluxed flow, curative craniotomy should be considered shortly after the TAE. A hybrid operating room equipped with an angiography system is suitable for the treatment of complex vascular malformations.

## Specific information for the patient

The patients presented in this study were healthy, without any known hereditary disorders or an obvious history of head trauma. In addition, there were no obvious neurological abnormalities before ictus.

## Supplementary information

Below is the link to the electronic supplementary material.

Supplementary file1 (MP4 1067335 KB)

## Data Availability

No datasets were generated or analysed during the current study.
